# Role of microRNAs in programmed cell death in renal diseases: A review

**DOI:** 10.1097/MD.0000000000033453

**Published:** 2023-04-14

**Authors:** Yan Zhang, Xinghua Lv, Feng Chen, Qian Fan, Yongqiang Liu, Zhanhai Wan, Janvier Nibaruta, Jipeng Lv, Xuena Han, Lin Wu, Hao Wang, Yufang Leng

**Affiliations:** a Department of Anesthesiology, First Hospital of Lanzhou University, Lanzhou, Gansu, China; b The First Clinical Medical College of Lanzhou University, Lanzhou, GanSu Province, China; c Tianjin Eye Hospital, Tianjin Key Lab of Ophthalmology and Visual Science, Tianjin Eye Institute, Nankai University Affiliated Eye Hospital, Tianjin, China; d Nankai Eye Institute, Nankai University, Tianjin, China; e Clinical College of Ophthalmology, Tianjin Medical University, Tianjin, China.

**Keywords:** apoptosis, autophagy, ferroptosis, microRNAs, programmed cell death, pyroptosis

## Abstract

MicroRNAs (miRNAs) regulate gene expression involving kidney morphogenesis and cell proliferation, apoptosis, differentiation, migration, invasion, immune evasion, and extracellular matrix remodeling. Programmed cell death (PCD) is mediated and regulated by specific genes and a wealth of miRNAs, which participate in various pathological processes. Dysregulation of miRNAs can disrupt renal development and induce the onset and progression of various renal diseases. An in-depth understanding of how miRNAs regulate renal development and diseases is indispensable to comprehending how they can be used in new diagnostic and therapeutic approaches. However, the mechanisms are still insufficiently investigated. Hence, we review the current roles of miRNA-related signaling pathways and recent advances in PCD research and aim to display the potential crosstalk between miRNAs and PCD. The prospects of miRNAs as novel biomarkers and therapeutic targets are also described, which might provide some novel ideas for further studies.

## 1. Introduction

MicroRNAs (miRNAs) are small endogenous non-coding RNAs with 18 to 24 nucleotides in length,^[1]^ which inhibit protein translation by interacting with target genes as epigenetic regulators. Current annotation in the miRBase database shows that the human genome contains 1917 annotated hairpin precursors and 2654 mature miRNAs that regulate more than 60% of human protein-coding genes, many of which are conserved in animals, and that these conserved miRNAs preferentially regulate most human mRNAs.^[2]^ It is estimated that more than 30% of the translation of encoded genes are regulated by miRNAs.^[3]^ Up to now, numerous studies have shown that miRNAs participate in cell development, differentiation, embryogenesis, metabolism, disease onset and development.^[3–5]^ Dysregulation of miRNA expression disrupts early kidney development and is implicated in the pathogenesis of hereditary and chronic renal diseases.^[6,7]^ Meanwhile, programmed cell death (PCD) play critical roles in the development of multicellular organisms and in the maintenance of human tissues, such as apoptosis, autophagy, pyroptosis, and ferroptosis. In contrast to transient, catastrophic, and uncontrollable unprogrammed death (e.g., necrosis), PCD is highly, tightly regulated and pharmacologically, genetically modulated.^[8,9]^ Thus, this review is written as follows: in Section 1, we discuss the current knowledge on miRNA biogenesis, function, and miRNA-target interaction; in Section 2, we discuss the crosstalk between microRNA and PCD in renal diseases and its clinical applications; in Section 3, we conclude through discussing the utility of miRNAs as potential promising biomarkers and therapeutic targets. And finally, we summarize the content and highlight critical questions for future research.

## 2. MicroRNA biogenesis, function, and miRNA-target interaction

**Figure 1.**
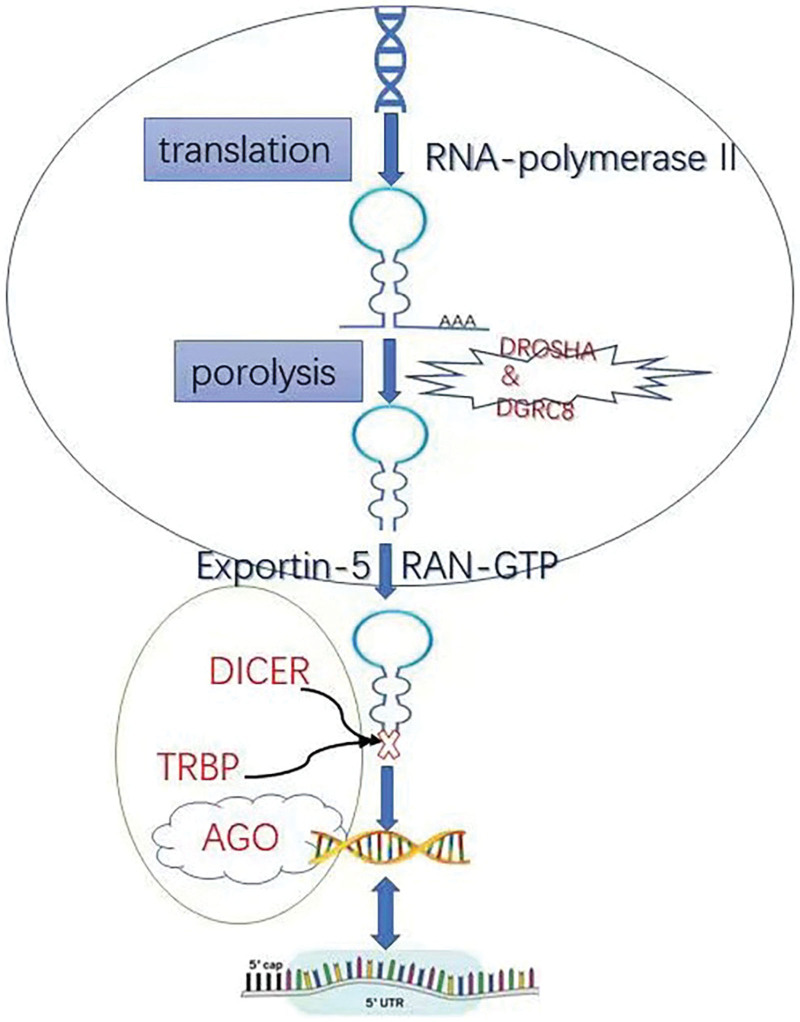
The overall mechanism of action regarding RNA biogenesis and function. MiRNA biogenesis occurs from the nucleus, where RNA polymerase II transcribes miRNA-encoding genes into hairpin transcripts with caps and polyadenosines. After transcription, the DROSHA in collaboration with the ribonuclease III microprocessor complex subunit DGRC8, cleaves the pri-miRNA into a 70-nucleotide hairpin structure known as pre-miRNA. Exportin-5 and the GTP-binding nuclear protein RAN (RAN-GTP) mediate the export of the pre-miR from the nucleus to the cytoplasm, where the pre-miRNA is processed by cleavage of its terminal loop by DICER and TAR RNA-binding protein (TRBP) (or TARBP2), eventually producing a 22-nucleotide miRNA duplex consisting of a guide and a guest strand (miRNA: miRNA, respectively). Then, the miRNA duplex is loaded onto the Argonaute (AGO) protein to form RISC. RISC consists of DICER, TRBP, and AGO1, and is subsequently directed toward the target mRNA, and if the seed sequence is highly conserved at positions 2 to 7 from the 50 end of the miRNA and has partial or full sequence complementarity with its target, then the miRNA binds to the 3′untranslated region (3′UTR) of the mRNA, leading to translational repression or mRNA degradation, respectively. The 5′-end domain of miRNAs, extending from nucleotide position 2 to 7, interacts with specific regions of the 3′UTR of their target mRNAs to induce translational repression and/or mRNA methylation and decay. miRNAs = microRNAs.

Generally, miRNA biogenesis occurs from the nucleus, where RNA polymerase II transcribes miRNA-encoding genes into hairpin transcripts with caps and polyadenosines. It is known as primary miRNAs or pri-miRNAs, which depend on their genomic location. MiRNA-encoding genes can be classified as intragenic (located within host gene introns) or intergenic, which have their own transcriptional regulatory elements. After transcription, the *DROSHA* in collaboration with the ribonuclease III microprocessor complex subunit *DGRC8*, cleaves the pri-miRNA into a 70-nucleotide hairpin structure known as pre-miRNA. Exportin-5 and the GTP-binding nuclear protein RAN mediate the export of the pre-miR from the nucleus to the cytoplasm, where the pre-miRNA is processed by cleavage of its terminal loop by DICER and TAR RNA-binding protein (or TARBP2), eventually producing a 22-nucleotide miRNA duplex consisting of a guide and a guest strand (miRNA: miRNA, respectively).^[10]^ Then, the miRNA duplex is loaded onto the Argonaute protein to form RISC. RISC consists of DICER, TAR RNA-binding protein, and argonaute1, among others, and is subsequently directed toward the target mRNA, and if the seed sequence is highly conserved at positions 2 to 7 from the 50 end of the miRNA and has partial or full sequence complementarity with its target, then the miRNA binds to the 3′ untranslated region (3′ UTR) of the mRNA, leading to translational repression or mRNA degradation, respectively.^[1]^ The 5′-end domain of miRNAs, extending from nucleotide position 2 to 7, interacts with specific regions of the 3′ UTR of their target mRNAs to induce translational repression and/or mRNA methylation and decay.^[11]^ However, miRNA binding sites have also been identified in other regions, including the 5’ UTR, coding sequences, and gene promoters.^[12]^ Although miRNAs are primarily associated with gene repression, post-transcriptional upregulation of miRNAs may also occur.^[13]^ The overall mechanism of action regarding RNA biogenesis and function is outlined in Figure 1.

MiRNAs biogenesis is under strict spatial and temporal control to ensure proper miRNA expression in response to various cellular signals. The regulation of miRNA biogenesis occurs at multiple levels, including binding of transcription factors to enhance miRNA genes, DROSHA processing of pri-miRNA, DICER processing of pre-miRNA, RNA methylation, editing of miRNA precursors, adenylation, uridylation, RNA decay, and many other mechanisms.^[13,14]^ Recently, super-enhancers have also emerged as a new class of regulatory elements to control miRNA biogenesis by increasing transcription and DROSHA/ DGCR8-mediated pri-miRNA processing. Combined with a broad *H3K4me3* profile, super-enhancer activity shapes the expression patterns and functions of tissue-specific miRNA.^[15]^ MiRNAs regulate kidney function by engaging the 3-UTR of downstream mRNAs and modulating mRNA transcription and translation. Recently, miR-20a-5p negatively regulates ACSL20 by targeting the 5`untranslated region of mRNA, thereby inhibiting ACSL4-dependent ferroptosis and attenuating acute kidney injury (AKI) triggered by renal ischemia-reperfusion injury. Moreover, in a systematic evaluation comparing CKD-associated miRNAs in general and high-risk subgroups of the population, investigators explored the specific expression patterns of identified miRNAs in prevalent CKD. Importantly, in the prediction signature, Koide et al discovered that the miRNAs transcriptome profile of circulating extracellular vesicle targeting VEGFA signaling as a Vascular Calcification (VC) factor in CKD. Overall, the biological features of RNA are inextricably linked to kidney disease.

## 3. MicroRNA crosstalk with PCD in renal diseases and clinical applications

### 3.1. Function of microRNA in autophagy

Autophagy is an important process that facilitates the maintenance of renal homeostasis and pathophysiology through lysosomal degradation to recycle damaged or excess organelles and protein aggregates. Dysregulation of autophagy involves in many chronic kidney diseases (CKDs), including diabetic nephropathy (DN), polycystic kidney disease, and focal segmental glomerulosclerosis.^[16–18]^ Autophagy is activated under stressful conditions and may serve as a protective response for the survival of renal fibrotic cells. The lack of autophagy and proteasome pathways leads to the development of glomerulosclerosis and proteinuria in senescent mice. Stimulation of senescence by D-gal treatment may trigger autophagy. During renal senescence, abnormally elevated levels of miR-155 attenuates D-gal-induced renal senescence and injury, which regulates PI3K/Akt signaling pathway-induced autophagy inhibition.^[19]^

DN is considered as the major concern for diabetic chronic microvascular complications and the most common cause of end-stage renal disease. Early renal hypertrophy is the main pathological feature, which gradually leads to glomerular extracellular matrix deposition and tubulointerstitial fibrosis, and eventually irreversible structural damage to the kidney. There is increasing evidence that miRNAs regulate the development of DN and may be involved in the regulation of fibrosis. MiR-22 expression was upregulated in DN rat kidney tissue. High glucose (HG)-treated NRK-52E cells could directly target gene phosphatases and tensin homologs (*PTEN*) to partially inhibit autophagy, which in turn promote tubulointerstitial fibrosis.^[20]^ Moreover, TGF-β and miR-192 reduce autophagy in mouse thylakoid cells under diabetic conditions, which can be reversed by inhibiting or deleting miR-192, indicating miR-192 as a useful therapeutic target for diabetic neuropathy.^[21]^ Recently, researchers found that silencing miR-150-5p promoted the interaction between SIRT1 and p53, leading to inhibition of p53 acetylation in podocytes, causing AMPK-dependent autophagy and acting as a renal function protector in DN mice.^[22]^ Similarly, miR-141-3p levels were increased in response to hyperglycemia, and overexpression of miR-141-3p exacerbated fibrosis and further inhibited autophagy in DN rats.^[23]^

MiR-20a may cause AKI in septic rats through activation of autophagy.^[24]^ In contrast, miR-214 can ameliorate sepsis-induced AKI through *PTEN/AKT/mTOR*-regulated autophagy.^[25]^ Ischemia/reperfusion injury (IRI) plays major role in renal transplantation and is one of the major causes of AKI or even renal graft failure. MiR-106b-5p was found probing the renal tissues of I/R-induced AKI rats and in hypoxia-reoxygenated (HR)-induced rat renal proximal tubular epithelial cells (NRK-52E). The results revealed that HR induction inhibited the proliferation of NRK-52E cells and promoted apoptosis and autophagy, while the miR-106b-5p antagonist promoted the proliferation under HR conditions and attenuated the apoptosis and autophagy of NRK-52E cells. Furthermore, the effects of miR-106b-5p antagonist on NRK-52E cell proliferation, apoptosis, and autophagy were mediated through the regulation of TCF4. Similarly, in vivo, miR-106b-5p antagonists reduced the severity of kidney injury, decreased cell proliferation in kidney tissue, and reduced serum creatinine (Scr) and blood urea nitrogen levels in IR-induced blood samples from AKI rats.^[26]^ IR also inhibited autophagic activity and increased miR-34a expression level in kidney tissue. MiR-34a can bind directly to the Atg4B 3’-untranslated region and knockdown of Atg4B expression inhibits autophagic activity in RTEC.^[27]^ A recent study found that miR-17-5p expression is upregulated when renal IRI occurs, inhibiting *PTEN* and *BIM* expression to suppress apoptosis and autophagy, which in turn upregulates downstream Akt/Beclin1 expression, providing a new possible therapeutic strategy for the prevention and treatment of renal IRI.^[28]^ MiR-30a-5p increased in the mouse renal I/R model and in the hypoxia-reoxygenation model of HK-2 and might attenuate autophagy by regulating the *Beclin-1/Atg16* pathway.^[29]^

Additionally, miR-30b inhibited VC and could curb progressive VC in CKDs hyperphosphatemia by enhancing autophagy. MiR-376b and direct target *Atg5* exhibited high and low expression in renal tissues of CKDs mice, which were treated with miR-376b inhibitors and exhibited reduced collagen deposition, inhibition of interstitial fibrosis, higher levels of autophagy, higher reactive oxygen species (ROS) production, enhanced apoptosis, and inhibition of kidney fibroblasts proliferation, suggesting that downregulation of miR-376b could have beneficial effects.^[30]^ The activation of the miR-4516/SIAH3/PINK1 mitochondrial autophagy signaling pathway maybe a viable new strategy for the treatment of CKDs^[31]^ (Table 1).

**Table 1:** MiRNAs crosstalk autophagy and correlated pathways are involved in renal diseases.

Disease	Model	MiRNAs	Expression	PCD function	Possible target	Clinical significance/observed effects
DN	vivo, vitro	MiR-22, MiR-141-3p	up	Suppress autophagy	Regulating the PTEN/Akt/mTOR pathway	Promoting Renal Tubulointerstitial Fibrosis
DN	vivo, vitro	MiR-192	up	Promote autophagy	Controlling TGF-beta-induced Col1a2 expression by down-regulating E-box repressors.	No data
DN	vivo, vitro	MiR-150-5p	up	Suppress autophagy	Targeting SIRT1/p53/AMPK Pathway	Silencing of miR-150-5p played a reno-protective role in DN mice through targeting SIRT1.
AKI	vivo	MiR-20a	up	Promote autophagy	No data	No data
AKI	vivo	MiR-214	up	Suppress autophagy	Regulating the PTEN/Akt/mTOR pathway	Ameliorating CLP-induced AKI
I/R	vivo, vitro	MiR-106b-5p	up	Promote autophagy	Upregulating expression of TCF4	Antagonist targeting miR-106b-5p attenuates acute renal injury
I/R	vivo, vitro	MiR-34a	up	Suppress autophagy	Targeting Atg4B	Causing injury in I/R RTECs
I/R	vivo, vitro	MiR-17-5p	up	Suppress autophagy	Inhibiting the PTEN and BIM pathways	Attenuating kidney IRI
I/R	vivo, vitro	MiR-30a-5p	down	Suppress autophagy	Regulating beclin-1/ ATG16 pathway	Moderating renal IRI
CKDs	vivo	MiR-376b	up	Suppress autophagy	Upregulating Atg5	Inhibition of microRNA-376b Protects Against Renal Interstitial Fibrosis
CKDs	vivo, vitro	MiR-4516	down	Promote autophagy	Targeting SIAH3/PINK1 mitophagy signaling axis	Activating miR-4516 can improve Renal Fibrosisbe

AKI = acute kidney injury, AMPK = AMP-activated protein kinase, CKDs = chronic kidney diseases, DN = diabetic nephropathy, I/R = ischemia/reperfusion, IRI = ischemia/reperfusion injury, miRNAs = microRNAs, PCD = programmed cell death, PINK1 = PTEN-induced kinase 1, PTEN = phosphatases and tensin homologs, SIAH3 = siah E3 ubiquitin protein ligase family member 3, SIRT1 = silencing information regulator 2 related enzyme 1.

### 3.2. Function of microRNA in pyroptosis

Pyroptosis is characterized by the formation of gasdermin protein family-mediated membrane perforation, cell collapse, and the release of inflammatory factors including IL-1β and IL-18, an important innate immune mechanism. Recently, the regulation of pyroptosis by miRNAs in human diseases and can be involved in the pathological process of renal diseases by acting directly or indirectly on proteins. MiR-155 expression was significantly increased in kidney tissues and the levels of pyroptosis-related proteins were also significantly increased. MiRNAs contained in exosomes of different origins have gradually surfaced. The greatest difference in miR-93-5p expression was observed in M1 and M2 macrophage-derived exosomes and that exosomal miR-93-5p could directly target thioredoxin-interacting protein to inhibit the pyroptosis pathway in renal tubular epithelial cells and attenuate sepsis-induced AKI.^[32]^ Macrophage-derived miR-155-containing exosomes promote cardiomyocyte pyroptosis and uremic cardiomyopathic changes, that is, cardiac hypertrophy and fibrosis, by directly targeting FoxO3a.^[33]^ MiR-4449 was upregulated in serum exosomes and miR-4449 levels increase with disease severity.^[34]^ MiR-21-5p in macrophage-derived extracellular vesicles regulate pyroptosis-mediated podocyte injury via A20 in DN.^[35]^

MiR-30c-5p was associated with NLRP3/cystatin-1 (caspase-1)-mediated activation of pyroptosis, and its overexpression alleviated renal injury. Importantly, TXNIP is a direct target of miR-30c-5p, and upregulation of miR-30c-5p suppressed TXNIP expression, which inhibited NLRP3, ASC and caspase-1 expression, and inflammatory cytokine secretion.^[36]^ Similarly, NLRP3-mediated SAKI pyroptosis is regulated by the long-stranded non-coding RNA (lncRNA) PVT1/miR-20a-5p axis, providing an important potential therapeutic target for septic AKI.^[37]^ Furthermore, downregulation of miR-223-3p can activate the NLRP3/Caspase-1/IL-1β pathway, exacerbating oxidative stress and pyroptosis during SAKI.^[38]^ TXNIP/NLRP3-mediated pyroptosis is involved in the development of septic AKI. Different miRNAs can regulate the development of DN by targeting NLRP3. TXNIP expression and stimulates NLRP3/TXNIP pathway-mediated pyroptosis and renal injury. Downregulating of IRE1α and upregulating miR-200a to suppress TXNIP reduces the occurrence of pyroptosis.^[39]^ Besides, miR-34c, as a target gene of lcnRNA NEAT1, can also mediate the effect of NEAT1 on DN cell pyroptosis by regulating NLRP3 expression as well as caspase-1 and IL-1β expression.^[40]^ Moreover, miR-23a-3p targeting NEK7 alleviates NLRP3-induced pyroptosis and liver and kidney injury in type 2 diabetic rats.^[41]^ MiR-200c is also involved in atorvastatin to protect podocytes from high-glucose-induced pyroptosis and oxidative stress, providing a potential target for DN. Specifically, overexpression of miR-200c resulted in elevated MALAT1 levels, diminished NRF2 levels, and reduced podocyte damage. It may be the mechanism by which atorvastatin podocytes are shielded from injury.^[42]^ More intriguingly, miR-1656 could increase ROS release by targeting *GPX4*, activating NLRP3 inflammatory vesicles, and releasing inflammatory factors IL-1β and IL-18 in selenium-deficient broiler kidney tissues^[43]^ (Table 2).

**Table 2:** MiRNAs crosstalk pyroptosis and correlated pathways are involved in renal diseases.

Disease	Model	MiRNAs	Expression	PCD function	Possible target	Clinical significance/observed effects
I/R	vivo, vitro	MiR-155	up	Promote pyroptosis	Inhibition of FoxO3a expression by the caspase recruitment domain (ARC) and its downstream protein apoptosis	Promoted cardiomyocyte pyroptosis and uremic cardiomyopathy changes (cardiac hypertrophy and fibrosis)
AKI	vivo, vitro	MiR-93-5p	No data	Inhibit pyroptosis	Exosomal miR-93-5p could directly target TXNIP to inhibit the pyroptosis pathway	Attenuating sepsis-induced AKI
DKD	vitro	MiR-4449	up	Promote pyroptosis	miR-4449 mimics targeting HIC1 promote HK2 cells to undergo pyroptosis	MiR-4449 is a cargo of serum exosomes in patients with DKD, and its levels increase as the disease progresses
DN	vivo, vitro	MiR-21-5p	up	Promote pyroptosis	Binding with its 3′-untranslated regions to inhibit A20	Inhibition of miR-21-5p in HG-treated macrophages EVs could inhibit inflammatory and alleviate podocyte injury
AKI	vivo	MiR-30c-5p	down	Inhibit pyroptosis	Suppressed TXNIP expression, which inhibited NLRP3, ASC and caspase-1 expression, inflammatory cytokine secretion	Overexpression of miR-30c-5p alleviated renal injury
AKI	vivo, vitro	MiR-20a-5p	down	Inhibit pyroptosis	Inhibited NLRP3	No data
AKI	vivo, vitro	MiR-223-3p	down	Inhibit pyroptosis	MiR-223-3p can activate the NLRP3/Caspase-1/IL-1β pathway	KLF6 inhibited miR-223-3p via binding to the miR-223-3p aggravate SAKI
DN	vivo, vitro	MiR-200a	down	Inhibit pyroptosis	MiR-200a to downregulate TXNIP	Upregulating miR-200a protects the kidneys
DN	vivo, vitro	MiR-34c	down	Inhibit pyroptosis	Targeting NLRP3 expression as well as caspase-1 and IL-1β expression	Inhibit fibrosis, apoptosis, and EMT in the kidney
DN	vivo, vitro	MiR-23a-3p	down	Inhibit pyroptosis	Targeting NEK7	Alleviated liver and kidney damage in type 2 diabetes mellitus rats
DN	vitro	MiR-200c	up	Promote pyroptosis	Targeting NRF2 signal pathway	No data

AKI = acute kidney injury, DKD = chronic kidney diseases, DN = diabetic nephropathy, HG = high glucose, I/R = ischemia/reperfusion, IRI = ischemia/reperfusion injury, miRNAs = microRNAs, NLRP3 = NOD-like receptor family pyrin domain containing 3, NRF2 = nuclear factor erythroid 2-related factor 2, PCD = programmed cell death, PTEN = phosphatases and tensin homologs, TXNIP = thioredoxin-interacting protein.

**Figure 2.**
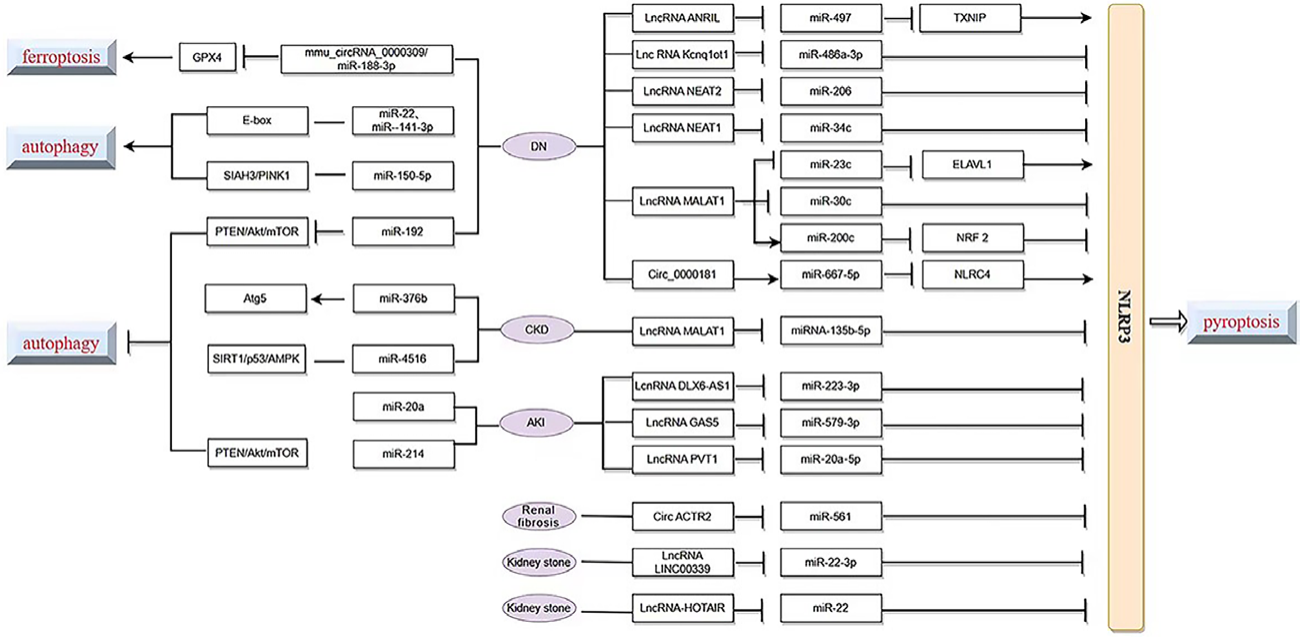
Non-coding RNA acts as miRNA sponge in pyroptosis, ferroptosis, and autophagy. This figure summarizes the miRNAs and concrete pathways corresponding to autophagy, pyroptosis and ferroptosis in PCD in renal diseases. miRNAs = microRNAs, PCD = programmed cell death.

Two other non-coding RNAs, such as lncRNA and circular RNA (circRNA), can act as miRNA sponges, competitively binding mRNAs involved in various biological processes. Circ_0000181 has been found to regulate miR-667-5p targeting NLRC4 to promote DN pyroptosis progression.^[44]^ Circ ACTR2 sponge miR-561 in macrophages by upregulating NLRP3 expression levels can induce IL-1β secretion and activate epithelial-mesenchymal transition to promote renal fibrosis.^[45]^ The subunit complement C5b-9 (sC5b-9) induces podocyte pyrogenesis by regulating the lncRNA Kcnq1ot1/miR-486a-3p/NLRP3 axis, providing more possibilities for podocyte-targeted treatment of inflammatory renal diseases.^[46]^ LncRNA MALAT1 competed with multiple miRNAs and competitively bound to multiple renal disease processes. LncRNA MALAT1 attenuates renal injury by acting as an RNA sponge for miRNA-135b-5p to regulate NLRP3, which is involved in regulating pyroptosis.^[47]^ LncRNA MALAT1 can also regulate miR-23c targeting ELAVL1 in DN to regulate renal tubular epithelial cell pyroptosis.^[48]^ LncRNA-ANRIL with spongy effects can directly target miR-497 to promote NLRP3 inflammatory vesicle activation and promote pyroptosis during DN.^[49]^ The lcnRNA DLX6-AS1 promotes LPS-induced pyroptosis in HK-2 cells by regulating the miR-223-3p/NLRP3 axis, and overexpression of miR-223-3p reverses this effect.^[50]^ Overexpression of LINC00339 and NLRP3 activated NLRP3 inflammatory vesicles and promoted pyroptosis in COM-treated HK-2 cells, whereas miR-22-3p mimics and NLRP3 knockdown exerted the opposite effect, that is, LINC00339 acts as a competitive endogenous RNA (ceRNA) by promoting NLRP3 expression through sponge miR-22-3p.^[51]^ Hyperuricemia has become one of the major risk factors for CKDs, which can cause renal microvascular disease, vascular endothelial dysfunction, and inflammatory cytokines release, and subsequently causing renal injury.^[52]^ LncRNA-HOTAIR has been shown to promote endothelial cell scorching by competitively binding miR-22 to regulate NLRP3 expression,^[53]^ which causes renal inflammation in hyperuricemic mice. LncRNA GAS5 inhibits miR-579-3p to activate the SIRT1/PGC-1α/Nrf2 signaling pathway and reduces cell scorching in sepsis-related kidney injury.^[54]^ LncRNA MALAT1 promotes HG-induced renal tubular epithelial cell pyroptosis by targeting NLRP3 through sponge miR-30c.^[55]^ LncRNA NEAT2 regulates HG-induced tubular cell pyroptosis in DN through miR-206.^[56]^ All of these are involved in the regulation of pyroptosis occurrence in kidney disease (Fig. 2).

### 3.3. Function of microRNA in ferroptosis

Unlike autophagy and pyroptosis, ferroptosis is an iron-dependent, peroxidation-driven, non-apoptotic form of PCD that is a process of enhanced iron accumulation and lipid peroxidation and is mainly characterized by the disruption of cellular antioxidant systems and elevated intracellular iron levels.^[57]^ Notably, ferroptosis participates in the development of severe inflammatory diseases and the abundance of ferritinases in cells promotes the release of inflammatory factors.^[58,59]^

HG-induced podocyte apoptosis is associated with mitochondrial damage in DN. As miRNA sponges, circRNAs work together with miRNAs to participate in regulating ferroptosis occurrence. DN mice had increased release of lipid ROS and decreased levels of GPX4 and GSH activity. Mmu_circRNA_0000309 could sponge miR-188-3p and reduce the expression of miR-188-3p. Meanwhile, *GPX4* was shown to be positively correlated with mmu_circRNA_0000309. Notably, miR-188-3p can also bind to the 3′ UTR of *GPX4* and negatively regulate its expression. Thus, mmu_circRNA_0000309 can competitively sponge miR-188-3p and then block its inhibitory effect on GPX4 expression, resulting in DN ferroptosis.^[60]^ Oxidative stress and ferroptosis eventually lead to the accumulation of ROS by causing regulatory cell injury and death. *GPX4* was shown to be a target of miR-214-3p. Inhibition of miR-214-3p enhanced the expression of *GPX4* and *SLC7A11*, while decreasing the expression of ACSL4, preventing cells from ferroptosis in renal tubular injury.^[61]^ These findings provide more possible prevention of AKI caused by cisplatin in future strategies. Enrichment in glycerophospholipid metabolism after metabolomic analysis, followed by increased expression of LPCAT3 metabolizing enzymes in SAKI patients, and bioinformatics analysis with TargetScanHuman was performed to confirm that miR-124-3p.1 could target the *LPCAT3* gene for negative feedback regulation. The main substrate for lipid peroxidation in ferroptosis is phospholipids containing PUFAs and is positively regulated by *LPCAT3.* MiR-124-3p.1/*LPCAT3*-induced ferroptosis is one of the pathogenic mechanisms of AKI.^[61]^

AKI is characterized by a sustained loss of renal function.^[62]^ Heme oxygenase-1 gradually decreased with increasing reoxygenation time, while miR-3587 expression was steadily upregulated, and administration of miR-3587 inhibitor enhanced HO-1 expression and inhibited HR-induced ferroptosis in NRK-52E cells.^[63]^ MiRNAs can also play their functional roles by cooperating with other non-coding RNAs. Multiple miRNAs can act synergistically in iron death in IRI. Among multiple miRNAs screened using databases, miR-182-5p and miR-378-3p could promote iron death in renal epithelial cells and were validated by mimics and inhibitors as well as in vivo mouse IR models. To further explore the regulatory mechanism of miR-182-5p and miR-378a-3p, bioinformatics assays via TargetScan revealed that miR-182-5p and miR-378a-3p targeted *GPX4* and *SLC7A11* in renal epithelial cells, respectively.^[64]^

### 3.4. Necroptosis-related miRNA

Necroptosis-related apoptosis is morphologically distinct from apoptosis, which is closely associated with inflammation^[65]^ and is characterized by rupture of the plasma membrane, leakage of cell contents, and swelling of organelles.^[66]^ Necroptosis is not dependent on cystathionine but is regulated through the involvement of multiple cellular signaling proteins, including receptor-interacting protein kinase 1, receptor-interacting protein kinase 3 (RIPK3) and its downstream target, and the mixed-spectrum kinase structural domain (MLKL).^[67]^ Many cellular mediators can activate necrotrophic apoptotic pathways such as death receptors, interferons, toll-like receptors and RNA or DNA sensors.^[67]^ TargetScan analysis showed that miR-194-5P, miR-338-3P, miR-500a-3P, and miR-577 had similar MLKL binding sites. Has-miR-500a-3P was significantly inhibited cisplatin-induced HK2 cell death through inhibiting MLKL phosphorylation and membrane translocation to suppress HK2 cell injury and inflammation.^[68]^ MiR-500a-3P could also be loaded into liposome delivery vectors to form miR-LIP and directly control the expression of RIPK3 and MLKL to reduce kidney injury. MiR-LIP significantly controlled the phosphorylation of MLKL. The results reveal the molecular mechanisms behind such AKI necrosis and the role of miRNAs in targeting the MLKL pathway, and further emphasize the potential advantages of liposomes as delivery vehicle for miRNA therapeutics.^[69]^ 3-chloro-1,2-propanediol (3-MCPD) is also a group of process-induced food contaminants with nephrotoxicity. 38 classical miRNAs and 40 novel miRNAs were shown to be differentially expressed in kidneys treated with 3-MCPD-dipalmitate. MiR-223-3p was significantly upregulated during 3-MCPD-dipalmitate-induced AKI. MiR-223-3p was able to inhibit RIPK3 by targeting the 3′ untranslated region of RIPK3 to inhibit RIPK3 expression and mitigate 3-MCPD-dipalmitate-induced AKI.^[70]^

The next-generation sequencing (NGS) was used to characterize miRNA differential expression patterns in established advanced DN models in Black and Tan Brachyury ob/ob (leptin-deficient mutant) mice. The results showed that 99 miRNAs were significantly increased, and no miRNAs were significantly decreased. Potential targets of 99 miRNAs suggested that inflammatory and immune processes, identified necroptosis specifications, and supported the renal tubular cell phenotype changes in the pathogenesis of DN.^[71]^ RIP1/RIP3 pathway is involved in the regulation of renal IRI induced cell necrosis.^[72]^ Recently, miR-124 was found to attenuate renal IRI by negatively regulating PARP1 through inhibiting the TNFα/RIP1/RIP3 pathway and rescued the miR-124.^[73]^ MiR-26a-5p alleviated apoptosis by inhibiting the expression of RIP1, RIP3, and MLKL in necrotizing apoptosis.^[74]^

MiR-381-3p is an inhibitor of TNF-induced apoptosis in various cancer cells. Overexpression of miR-381-3p could inhibit the activation of RIPK3 and MLKL, then blocking TNF-induced necroptosis. Notably, the overall survival of RCC patients with high miR-381-3p expression was lower than that of patients with low miR-381-3p expression levels. MiR-381-3p is treated as a biomarker to predict the onset of necroptosis and a possible therapeutic target for RCC.^[75]^ Recently, a novel miRNA signature of the necroptosis phase for predicting the prognosis of clear cell renal cancer was reported, and after a series of assays and analyses, 6 necroptosis-related miRNAs in the risk profile were finally explored (has-miR-101-3p, has-miR-193a-3p, has-miR-200a-5p, has-miR-214-3p, has-miR-221-3p, has-miR-223-3p).^[76]^ (Table 3)

**Table 3:** MiRNAs regulate ferroptosis and necroptosis in renal diseases.

Disease	Model	MiRNAs	Expression	PCD function	Possible target	Clinical significance/observed effects
AKI	vivo, vitro	MiR-214-3p	up	Promote ferroptosis	Targeted GPX4 and SLC7A11	Inhibiting miR-214-3p would alleviate TEC ferroptosis in cis-AKI
AKI	vitro	MiR-124-3p	down	Inhibit ferroptosis	Target the LPCAT3	Suppresses cell proliferation by disrupting phospholipid metabolism
I/R	vitro	MiR-3587	up	Promote ferroptosis	Target HO-1	Inhibition of miR-3587 protects renal tissues
I/R	vivo, vitro	MiR-182-5p and MiR-378a-3p	up	Promote ferroptosis	Targeted GPX4 and SLC7A11	Silencing miR-182-5p and miR-378a-3p alleviated the I/R-induced renal injury in rats
AKI	vitro	Has-miR-500a-3P	down	Inhibit necroptosis	Binds to the 3′UTR of MLKL	Alleviates kidney injury
AKI	vivo, vitro	MiR-223-3p	up	Inhibit necroptosis	Targeting the 3’ un-translated region of RIPK3	Alleviates kidney injury
I/R	vivo, vitro	MiR-124	down	Inhibit necroptosis	Negatively regulating PARP1 to inhibit TNFα/RIPK1/RIPK3 Pathway	Alleviate Renal Ischemia-reperfusion Injury
AKI	vivo	MiR-26a-5p	down	Inhibit necroptosis	Targeting PTEN to block PI3K/ AKT signaling pathway	No data

AKI = acute kidney injury, GPX4 = glutathione peroxidase 4, I/R = ischemia/reperfusion, IRI = ischemia/reperfusion injury, LPCAT3 = lyso-phosphatidylcholine acyltransferase-3, miRNAs = microRNAs, MLKL = mixed-spectrum kinase structural domain, PCD = programmed cell death, PTEN = phosphatases and tensin homologs, RIPK1 = receptor-interacting protein kinase 1, RIPK3 = receptor-interacting protein kinase 3.

## 4. Potential clinical application of miRNAs

As FDA-approved small RNA drugs begin to enter clinical medicine, ongoing research on miRNA-like small RNAs has surged their preclinical and clinical research applications. The first small interfering RNA was recently approved by the FDA in 2018. Patisiran, is cleared for the treatment of rare polyneuropathies caused by hereditary transthyretin-mediated amyloidosis and works by binding and degrading transcripts of transthyretin messenger RNAs. Numerous reports have demonstrated the remarkable utility of miRNAs as biomarkers of pathogenic diseases, modulators of drug resistance. Small molecule drugs have been successfully designed pharmacologically to target multiple targets or pathways, paving the way for miRNAs as future therapeutic approaches.^[77]^

### 4.1. Promising biomarkers for diagnosis and prognosis

Due to the abundance and accessibility of miRNAs, they are expected to be ideal biomarkers for the diagnosis and prognosis of renal diseases.^[78]^ These circulating miRNAs are packaged into particles (exosomes, microvesicles and apoptotic bodies).^[79]^ Under various storage conditions, miRNAs are relatively stable in serum and urine.^[80]^ However, the stability of exosomal miRNAs isolated from plasma depends on the method of exosome isolation and storage.^[81]^ Exosomal miRNAs appear to be more stable than non-exosomal miRNAs and are therefore preferred for biomarker studies.^[82]^ However, they rely on the availability and accurate annotation of miRNA sequences in databases for probe and primer design. NGS allows the simultaneous detection of known and novel miRNA species and provides high sensitivity.^[83]^ The single nucleotide resolution of NGS enables the identification of isoforms, which are mature miRNA isoforms that differ from canonical isoforms in length, sequence, or both, which alters the targeting specificity of miRNAs.^[84]^

Several studies have evaluated urinary and serum miRNAs in type 1 and type 2 DM patients in relation to different DKD stages. Eighteen urinary miRNAs were found to be strongly associated with the subsequent occurrence of microalbuminuria, while 15 miRNAs exhibited gender-related expression differences. The predicted targets of these miRNAs involved in the pathogenesis and progression of DN.^[85]^ MiR-15b, miR-34a, and miR-636 were upregulated in both glomeruli and exosomes of patients with type 2 DKD. There was a positive correlation between these miRNAs, serum creatinine, and urinary protein creatinine ratios.^[86]^

30 urinary exosomal miRNAs were identified as biomarkers in patients with pediatric nephrotic syndrome (NS). And the significant positive correlation between miR-194-5p and miR-23b-3p concentrations and urinary protein levels could distinguish these patients from healthy controls in terms of accuracy, sensitivity, and specificity.^[87]^ In addition, 5 different serum miRNAs and urinary miR-30a-5p were reported in pediatric NS, which may represent potential diagnostic or prognostic biomarkers for idiopathic pediatric NS.^[88]^

### 4.2. miRNAs-related therapeutic strategies

It has been proven that majority of miRNAs, while acting as biomarkers, are also potential therapeutic targets for many kidney diseases. MiR-205 and miR-206 serve as therapeutic targets for hypertensive nephropathy-induced injury, and miRNA-377 and miRNA-29a may be targeted as kidney tubular cells-derived conditioned medium to prevent diabetes-induced kidney injury. Oligonucleotide manipulation techniques, including anti-aging vectors, have been widely used to inhibit miRNA binding to mRNA in research settings or clinical trials.^[89]^ The main approaches for miRNA therapy include the use of miRNA mimics to restore miRNA levels or the use of anti-aging vectors to inhibit specific miRNAs.^[90]^ One of the challenges associated with the development of miRNA-based therapeutics is the identification of miRNA candidates for each disease. To narrow down candidate miRNAs for therapeutic intervention, patient samples should be carefully analyzed and combined with in vitro and in vivo assays to address the pathophysiological mechanisms.^[91]^ Another challenge includes in vivo stability and site-specific drug delivery with minimal toxicity and off-target effects. RNA molecules were modified with chemical modifications (2′-O-methyl, phosphorothioate or locked nucleic acids) in their backbone to provide greater stability and protection against nucleases found in serum or endocytic compartments.^[92]^ Delivery strategies using various delivery methods (intraperitoneal, intravenous, and subcutaneous) or vectors containing kidney-specific and inducible promoters have been used successfully for selective renal targeting.

## 5. Conclusions

MiRNAs work as novel biomarkers and therapeutic targets involving in miRNA-regulated PCD in renal diseases. To screen more effective therapeutic regimens, with the development of candidate miRNAs begin to initiate and complete potential pre-clinical trials, the future of diagnostic and potential application for specific renal disease treatment will arguably continue to thrive. Moving forward, with the groundbreaking advances of the Human Genome Project, emerging miRNA therapies are offering new hope for health conditions where current treatment options remain elusive. However, miRNA transcripts are significantly larger than protein-coding genes, and the road to future clinical trials is arduous. The identification of miRNA binding sites in target genes and their corresponding associated biological pathways by developing bioinformatics programs will facilitate the accelerated translation of miRNAs into clinical medicine.

### 5.1. Outstanding questions

We review and address current roles of miRNA-related signaling pathways and recent related advances in PCD research, with an emphasis on the potential crosstalk between miRNAs and PCD in renal morphological alterations and disease development. Notably, the roles of the pathways in different renal diseases are being in a context dependent manner. Moreover, new clues and prospects of miRNAs as potential new biomarkers and promising therapeutic targets in clinical applications are also described.

### 5.2. Search strategy and selection criteria

Content for this Review were identified by searches of PubMed and MEDLINE, and references from relevant articles using the search terms “microRNAs,” “programmed cell death,” “renal diseases,” “pathway,” “autophagy,” “pyroptosis,” “ferroptosis,” “apoptosis,” “biogenesis,” “necroptosis,” “diabetic nephropathy,” “membranous nephropathy,” “acute kidney injury,” “acute renal failure” and so on. Only articles published in English between 1991 and 2023 were included.

## Author contributions

**Conceptualization:** Yan Zhang.

**Data curation:** Yan Zhang, Qian Fan.

**Formal analysis:** Qian Fan, Yongqiang Liu, Zhanhai Wan.

**Investigation:** Yan Zhang, Xinghua Lv, Feng Chen, Zhanhai Wan, Janvier Nibaruta, Jipeng Lv, Xuena Han.

**Methodology:** Xinghua Lv, Janvier Nibaruta, Jipeng Lv, Xuena Han, Lin Wu.

**Resources:** Feng Chen, Qian Fan, Yongqiang Liu, Lin Wu, Hao Wang.

**Software:** Xinghua Lv, Feng Chen, Hao Wang.

**Supervision:** Yufang Leng.

**Validation:** Xinghua Lv, Yufang Leng.

**Writing – original draft:** Yan Zhang.

**Writing – review & editing:** Qian Fan, Yufang Leng.
